# DYNAMIC BENEFITS OF FINDING THE SILVER LINING: COUPLED CHANGES IN SECONDARY CONTROL AND WELL-BEING DURING COVID-19

**DOI:** 10.1093/geroni/igad104.2662

**Published:** 2023-12-21

**Authors:** Matthew Pierce, Jeremy Hamm, Katherine Duggan, Odalis Garcia

**Affiliations:** North Dakota State University, Fargo, North Dakota, United States; North Dakota State University, Fargo, North Dakota, United States; North Dakota State University, Fargo, North Dakota, United States; North Dakota State University, Fargo, North Dakota, United States

## Abstract

Secondary control beliefs (e.g., “negative experiences can be blessings in disguise”) reflect a psychological resource that can protect well-being for individuals who encounter uncontrollable life circumstances. However, previous research has focused on between-person differences in secondary control; little is known about the role of dynamic, within-person shifts in secondary control during major life stressors such as the COVID-19 pandemic. Our study examined whether within-person changes in secondary control beliefs were associated with corresponding within-person shifts in mental health and well-being during the pandemic. We analyzed 1-year data from the NDSU National COVID Study which contains 4 waves of data from a nationally representative sample of U.S. adults aged 18-80 (n = 293). Multilevel models assessed the extent to which within-person shifts in secondary control predicted corresponding changes in mental health (perceived stress, depressive symptoms), hedonic well-being (positive and negative affect, life satisfaction), and eudemonic well-being (personal growth, meaning and purpose). All models controlled for age, sex, education, income, and between-person differences in secondary control. Within-person changes in secondary control predicted corresponding shifts in mental health and well-being outcomes (bs = |.10-.25|, p < .005) except life satisfaction (b = .10, p = .061). In other words, participants reported increased mental health and well-being on occasions during the pandemic when they experienced increased secondary control. Findings advance the literature by documenting that secondary control exhibits dynamic shifts during a major life stressor. Additionally, these findings point to the adaptive role of such shifts (within-person increases) in supporting well-being during the pandemic.

